# Spatiotemporal Pattern and Its Determinants for Newly Reported HIV/AIDS Among Older Adults in Eastern China From 2004 to 2021: Retrospective Analysis Study

**DOI:** 10.2196/51172

**Published:** 2024-02-13

**Authors:** Gang Huang, Wei Cheng, Yun Xu, Jiezhe Yang, Jun Jiang, Xiaohong Pan, Xin Zhou, Jianmin Jiang, Chengliang Chai

**Affiliations:** 1 Health Science Center Ningbo University Ningbo China; 2 Department of AIDS and STD Prevention and Control Zhejiang Provincial Center for Disease Control and Prevention Hangzhou China; 3 Key Lab of Vaccine Prevention and Control of Infectious Disease of Zhejiang Province Hangzhou China

**Keywords:** HIV/AIDS, men who have sex with men, newly reported infections, older adults, spatiotemporal analysis

## Abstract

**Background:**

In recent years, the number and proportion of newly reported HIV/AIDS cases among older adults have increased dramatically. However, research on the pattern of temporal and spatial changes in newly reported HIV/AIDS among older adults remains limited.

**Objective:**

This study analyzed the spatial and temporal distribution of HIV/AIDS cases and its influencing factors among older adults in Eastern China from 2004 to 2021, with the goal of improving HIV/AIDS prevention and intervention.

**Methods:**

We extracted data on newly reported HIV/AIDS cases between 2004 and 2021 from a case-reporting system and used a Joinpoint regression model and an age-period-cohort model to analyze the temporal trends in HIV/AIDS prevalence. Spatial autocorrelation and geographically weighted regression models were used for spatial aggregation and influence factor analysis.

**Results:**

A total of 12,376 participants with HIV/AIDS were included in the study. The newly reported HIV infections among older adults increased from 0.13 cases per 100,000 people in 2004 to 7.00 cases per 100,000 people in 2021. The average annual percent change in newly reported HIV infections was 28.0% (95% CI –21.6% to 34.8%). The results of the age-period-cohort model showed that age, period, and cohort factors affected the newly reported HIV infections among older adults. The newly reported HIV/AIDS cases among men who have sex with men (MSM) had spatial clustering, and the hotspots were mainly concentrated in Hangzhou. The disposable income of urban residents, illiteracy rate among people aged 15 years or older, and number of hospital beds per 1000 residents showed a positive association with the newly reported HIV infections among older MSM in the Zhejiang province.

**Conclusions:**

HIV/AIDS among older adults showed an increasing trend and was influenced by age, period, and cohort effects. Older MSM with HIV/AIDS showed regional clustering and was associated with factors such as the disposable income of urban residents, the illiteracy rate among people aged 15 years or older, and the number of hospital beds per 1000 people. Targeted prevention and control measures are needed to reduce HIV infection among those at higher risk.

## Introduction

The first case of AIDS from HIV was reported in 1981 [1]. Since then, over 84 million individuals have been infected with HIV with over 40 million reported deaths attributed to AIDS-related illnesses. According to the Joint United Nations Programme on HIV/AIDS, approximately 38.4 million individuals are living with HIV, with 1.5 million new infections in 2021 [2]. In China, the absolute number of new HIV diagnoses has increased annually since 2005, reaching nearly 131,671 in 2020. By the end of 2020, a total of approximately 1,053,000 cases had been reported in China [3].

Initially, the epidemic was thought to affect primarily individuals aged between 15 and 49 years; however, evidence suggests that the burden of HIV/AIDS is considerable among those aged 50 years or older [4]. Epidemiological studies in China have also reported an increasing trend in the number and proportion of HIV infections among older adults [5]. A study from the Henan Province reported that the proportion of newly reported cases aged 50 years or older increased gradually from 4.5% between 1995 and 2000 to 35.5% between 2016 and 2020 through sexual transmission [6]. In China, the sexual needs of older adults are often neglected by their partners and society, often leading to high-risk sexual behavior. Unprotected sexual contact is very common among older people, which in turn increases the risk of HIV infection [4].

The generation, transmission, and distribution of HIV/AIDS are closely associated with geospatial information. Spatiotemporal analysis is commonly used in HIV/AIDS research to assess long-term trends and geographic distribution patterns [7-9]. The Joinpoint regression model can estimate long-term trends in reported HIV/AIDS incidence or infections, whereas the age-period-cohort model can estimate the effects of age, period, and cohort on HIV/AIDS annual reported incidence or infections [7]. Spatial analysis provides insights into the spatial distribution of the HIV/AIDS epidemic and risk factors associated with the geographically weighted regression (GWR) model [8]. For example, using AIDS intensity ranking maps and spatial cluster analysis, Wang et al [9] found that AIDS incidence hotspots in China in 2008 and 2011 were mainly distributed in Yunnan, Guangxi, Guizhou, Chongqing, and Sichuan.

In terms of factors influencing the AIDS epidemic, economic development is strongly positively associated with the AIDS epidemic in China; the GWR results further indicate that the impact of health care and education on the AIDS epidemic varies across regions [9]. However, the long-term trends and spatial distribution of HIV/AIDS prevalence among older adults in Eastern China have not been adequately assessed. The design and evaluation of national HIV/AIDS planning often rely on comprehensive national data, which can mask local HIV/AIDS epidemics [10]. To better control the HIV/AIDS epidemic, a more comprehensive and detailed analysis of HIV/AIDS among older adults in Eastern China is needed.

Therefore, this study assessed the changing trend and spatial distribution of newly reported HIV infections among older adults in Eastern China and aimed to clarify the temporal trends, hotspot areas, and influencing factors of the HIV/AIDS epidemic. Our findings provide novel insights into the developmental trend of the HIV/AIDS epidemic among older adults in Eastern China, help optimize resource allocation, and facilitate HIV/AIDS prevention and treatment strategies.

## Methods

### Ethical Considerations

This study was approved by the ethics review committee of the Center for Disease Control and Prevention of Zhejiang Province (2018-032). All of the surveillance work was completed by the local Centers for Disease Control and Prevention (CDCs). We extracted data from the Zhejiang Province database of the National Data Information System for Comprehensive HIV/AIDS Control. We did not collect extra information or specimens for this study. Therefore, our study is based on data derived from daily work and was exempt from informed consent. During data analysis, we strictly followed the requirements of the ethical review committee of the Zhejiang Provincial CDC to protect the privacy of the participants, and all data used in this study were anonymous.

### Study Area

The Zhejiang Province, located on the southern flank of the Yangtze River Delta along the southeast coast of China (latitude: N 27° 02'-31° 11' and longitude: E 118° 01'-123° 10'; Multimedia Appendix 1), has a land area of 105,500 km^2^ with approximately 65.77 million residents as of 2022 [11]. Currently, Zhejiang Province has 11 prefecture-level cities, 37 municipal districts, 20 county-level cities, and 33 counties.

### Data Sources

Since 2004, China has used a direct network reporting system called the China Infectious Disease Reporting Information System. The database was established by the Chinese CDC. Patients aged 50 years or older with newly reported HIV infection between January 1, 2004, and December 31, 2021, were screened from the Zhejiang Province database of the National Data Information System for Comprehensive HIV/AIDS Control. There were no significant differences in the data information system during the study period. In this study, patients with HIV/AIDS were diagnosed according to the HIV/AIDS diagnostic principles and China’s National HIV/AIDS Testing Technical Specifications and Standards [12].

Data on sociodemographic characteristics, transmission routes, and addresses were collected by local CDC staff in face-to-face interviews using standardized forms. Vectorized county-level geographic maps of the Zhejiang Province were derived from the National Geographic Information Public Service Platform. The area codes were determined by the Disease Control Information System.

To explore the factors influencing the newly reported HIV/AIDS cases according to geographical distribution, we collected the following variables: disposable income of urban residents, gross domestic product (GDP) per county, proportion of the illiterate population, proportion of the unmarried population, area of residence per capita, car ownership rate, number of hospital beds per 1000 people, number of doctors per 1000 people, urbanization rate, and proportion of the older population. Data were compiled using a Microsoft Excel (version 16.0; Microsoft Corporation) spreadsheet. Data on these influencing factors were obtained from the Statistical Yearbook of Zhejiang Province due to the presence of multicollinearity between the variables. The ordinary least squares (OLS) method was used to exclude redundant variables (variance inflation factor >7.5) and those that did not pass the significance test.

### Statistical Analysis

#### Temporal Analysis

The temporal analysis used the crude rate of newly reported HIV/AIDS for each year. The new reporting rate is calculated by dividing the total number of incidents in the year by the number of people in the corresponding year and multiplied by 100,000.

The annual percent change (APC) and average APC (AAPC) of each component were estimated using a Joinpoint regression model. The infection of new reports (*y_i_*) was set as the dependent variable, year was set as the independent variable (*χ_i_*), and sex and route of infection were set as subgroup variables [13]. Joinpoint regression analysis was performed using JPR software (version 4.9.1.0; Statistical Research and Applications Branch, National Cancer Institute).

The age-period-cohort model, a popular statistical tool for extracting information hidden in morbidities, has been used in long-term trend studies on social change, causes of disease, aging, demographic processes, and dynamic studies [7]. The effects of age, period, and cohort factors on the newly reported HIV infections were analyzed using an R language–based network analysis tool developed by the National Cancer Institute [14].

The model required consistent age, period, and cohort intervals; we divided the study period into four 5-year intervals, divided age into 7 groups, and calculated birth cohort by subtracting the period from age. The age, period, and cohort of the center were selected as controls [14].

#### Spatial Analysis

ArcGIS software (version 10.8; ESRI Inc) was used to map the spatial distribution of HIV/AIDS cases. The Global Moran I Index was applied to examine the clustering of HIV/AIDS among older adults at the county level in Zhejiang Province to determine whether the clustering elements within the region were statistically significant at α=.05 test level [15].

Local spatial autocorrelation was used to analyze the correlation of HIV/AIDS case distribution at the county level. In the Local Indicators of Spatial Autocorrelation (LISA) map, we identified 4 clusters of spatial relationships for the analyzed variables. High-high and low-low clusters are those in which the high or low values of the study variable are surrounded by neighboring areas above or below the mean [16].

The OLS model requires the data to be independently distributed, and the results are global estimates of the parameters that do not reflect the pattern of change in the data with geographical location. The GWR model embeds the spatial location of the data into regression parameters and uses local weighted least squares to estimate the parameters, which is a local statistical model [17]. In the presence of spatial autocorrelation, the traditional OLS model is not applicable to data analysis. In the GWR model, the region-specific regression coefficient is no longer the same value estimated using global information but rather a variable coefficient that varies with geographical location. The *R*^2^, adjusted *R*^2^, and corrected Akaike information criterion values were used to compare the OLS and GWR models [18]. The GWR model is structured as follows:



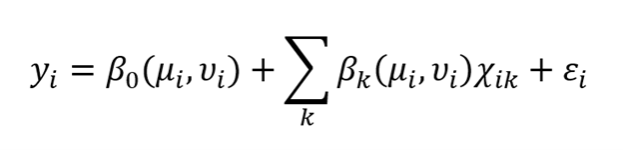



where (*μ_i_*, *ν_i_*) is the coordinate of the geographic center of the *i*th sample space unit and *β_k_* (*μ_i_*, *ν_i_*) is the value of the continuous function *β_k_* (*μ*, *ν*) in the space of the *i*th sample unit.

### Result

#### Demographic Characteristics

**Figure 1:**
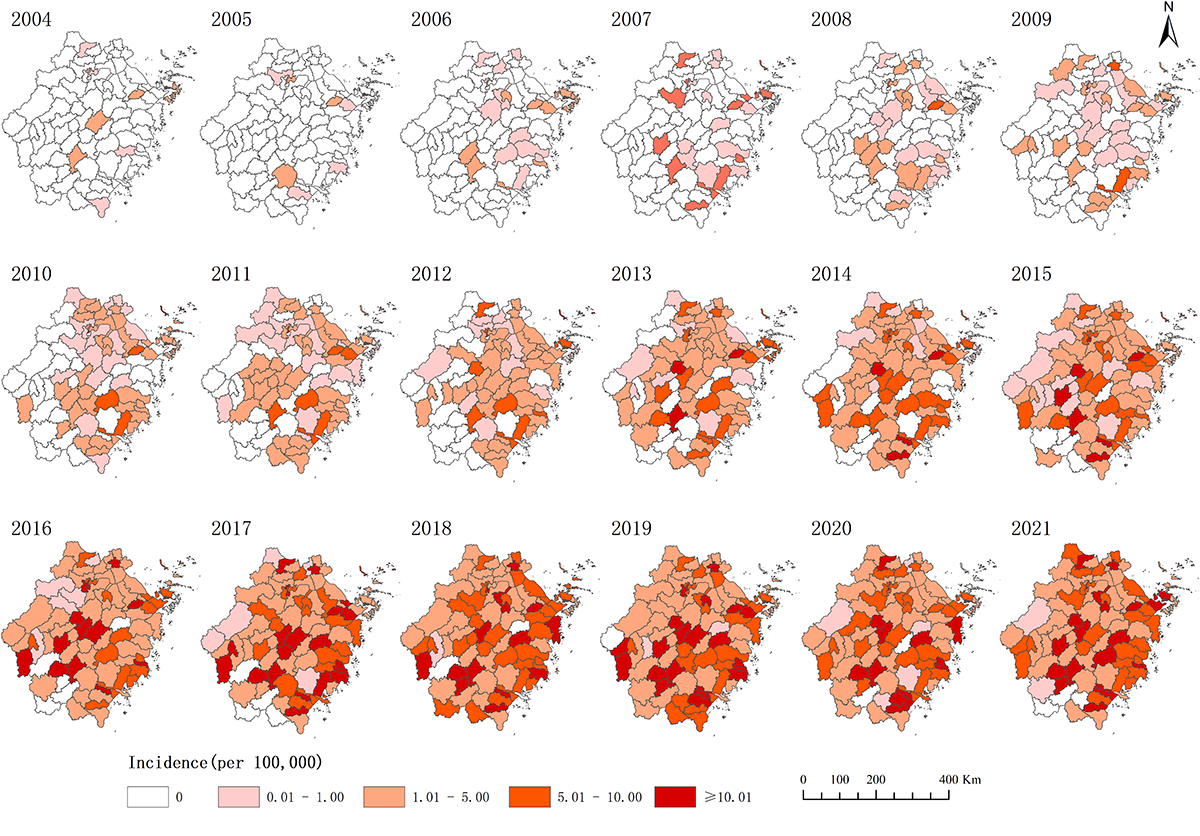
Distribution patterns of reported HIV/AIDS prevalence among older adults in the Zhejiang Province (2004-2021).

Between 2004 and 2021, a total of 12,376 new HIV/AIDS cases among older adults were reported in Zhejiang Province. The number of reported cases increased from 15 in 2004 to 1657 in 2021, and the number of reported infections increased from 0.13 per 100,000 persons in 2004 to 7.00 per 100,000 persons in 2021. In terms of geographical distribution, HIV/AIDS cases were reported in all counties and districts during the study period. In 2004, a total of 9 counties reported HIV/AIDS cases among older adults, while 86 newly reported HIV/AIDS cases were reported among older adults in 2021 (Figure 1). Of the 12,376 patients, 76.9% (9514/12,376) were male and 98.5% (12,196/12,376) of cases involved transmission through sexual contact (10,496/12,376, 84.8% heterosexual transmission, and 1700/12,376, 13.7% homosexual transmission; Table 1).

**Table 1:** Demographic characteristics of HIV/AIDS among older adults in Zhejiang Province (2004-2021).

Year	Reported cases, n	Male, n					Female, n		
		Heterosexual transmission	Homosexual transmission	Blood transmission	Other routes or unknown	Heterosexual transmission		Blood transmission	Other routes or unknown
2004	15	7	0	0	0	6		2	0
2005	11	6	0	2	0	0		2	1
2006	39	28	2	0	3	6		0	0
2007	51	31	1	4	3	11		1	0
2008	86	54	8	5	2	15		2	0
2009	164	107	15	1	5	32		1	3
2010	264	165	30	9	3	52		2	3
2011	318	196	48	2	4	62		3	3
2012	409	254	56	1	0	96		2	0
2013	561	364	85	1	3	107		0	1
2014	729	470	107	0	2	149		0	1
2015	838	538	133	1	0	166		0	0
2016	1129	710	142	3	3	270		0	1
2017	1454	965	167	2	9	307		0	4
2018	1502	932	202	2	7	357		0	2
2019	1685	1022	235	4	15	405		0	4
2020	1464	866	215	0	10	367		1	5
2021	1657	971	254	0	22	402		0	8
Overall	12376	7686	1700	37	91	2810		16	36

#### Temporal Trend and Joinpoint Regression Analysis

**Figure 2:**
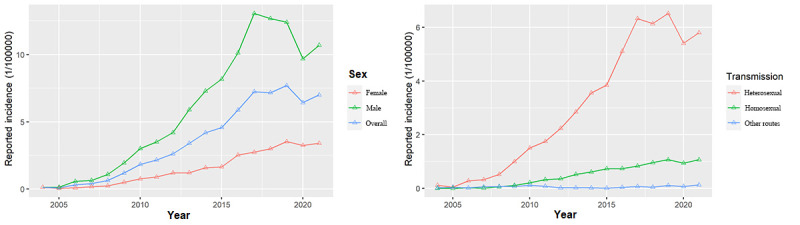
Trends in the reported HIV/AIDS infections among older adults by sex and transmission.

The newly reported HIV infections in older male individuals, which peaked in 2017 and then fluctuated downward from 2018 to 2021, were significantly higher than those in older female individuals in Zhejiang Province from 2004 to 2021. Heterosexual and homosexual transmission were the predominant routes of HIV/AIDS transmission among older adults, with the number of heterosexual transmission cases growing rapidly over the study period, reaching its highest recorded level in 2019. In contrast, the number of men who have sex with men (MSM) and who are HIV positive grew slower over the study period (Figure 2).

The results of the Joinpoint regression model for the newly reported HIV infections showed that the optimal results of the model were all 2 nodes. There was a significant increase in newly reported HIV infections among older adults in Zhejiang Province from 2004 to 2021 (AAPC=28.0%, 95% CI –21.6% to 34.8%; Table 2).

**Table 2:** Temporal trend analysis of newly reported HIV infections among older adults in Zhejiang Province (2004-2021).

Variables and segment end points (lower-upper)				Annual percent change (95% CI)	Average annual percent change (95% CI)			Trend segment	
**Overall**						28.0 (21.6 to 34.8)			3
	2004-2010			61.1 (37.9 to 88.2)					
	2010-2017			22.0 (17.2 to 27.1)					
	2017-2021			–1.3 (–5.8 to 3.4)					
**Sex**									
	**Male**					27.6 (19.8 to 36.0)			3
		2004-2010	62.0 (33.5 to 96.6)						
		2010-2017	23.7 (18.0 to 29.8)						
		2017-2021	–5.8 (–10.8 to –0.5)						
	**Female**					26.6 (18.5 to 35.2)			3
		2004-2010	51.5 (24.5 to 84.3)						
		2010-2018	20.1 (14.8 to 25.7)						
		2018-2021	1.6 (–8.1 to 12.4)						
**Routes of transmission**									
	**Heterosexual transmission**					28.7 (20.8 to 35.2)			3
		2004-2010	60.0 (34.8 to 89.9)						
		2010-2017	23.0 (17.7 to 28.6)						
		2017-2021	–2.5 (–7.3 to 2.5)						
	**Homosexual transmission**					42.4 (23.2 to 64.5)			3
		2004-2010	111.3 (34.0 to 233.1)						
		2010-2015	25.8 (12.7 to 40.4)						
		2015-2021	6.4 (2.7 to 10.2)						
	**Other routes or unknown**					10.2 (–15.3 to 43.3)			3
		2004-2010	29.9 (0.1 to 68.6)						
		2010-2013	–42.0 (–88.0 to 179.8)						
		2013-2021	23.9 (8.9 to 40.8)						

From 2004 to 2017, the newly reported infections showed an increasing trend (2004-2010: APC=61.1%, 95% CI –37.9% to 88.2%; 2010-2017: APC=22.0%, 95% CI –17.2% to 27.1%). However, there was no significant difference in the trend in newly reported infections from 2017 to 2021 (2017-2021: APC=1.6%, 95% CI –8.1% to 12.4%). Similar changes were observed in older male and female individuals, with AAPCs of 27.6% (95% CI –19.8% to 36.0%) and 26.6% (95% CI –18.5% to 35.2%) for older male and female individuals, respectively. For the newly reported infections of different routes of transmission, both heterosexual and homosexual transmission showed an increasing trend, with AAPC rates of 28.7% (95% CI –20.8% to 35.2%) and 42.4% (95% CI –23.2% to 64.5%), respectively. However, the annual trend for the other transmission routes was not significant, with an AAPC of 10.2% (95% CI –15.3% to 43.3%).

#### Age-Period-Cohort Model Analysis

#### Wald Chi-Square Test Results

**Figure 3:**
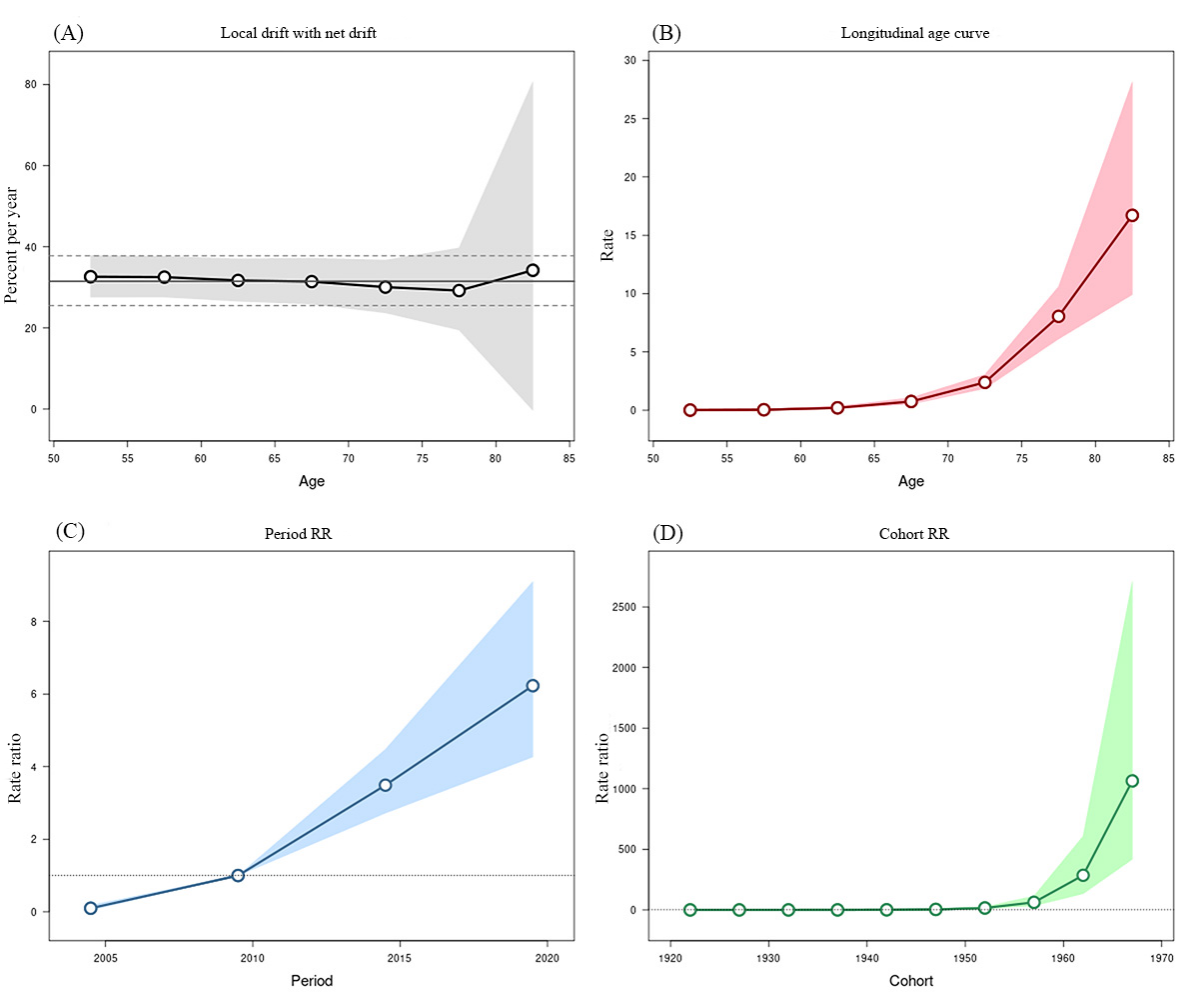
Age-period-cohort effect of reported HIV/AIDS infections among older adults in Zhejiang Province (2004-2021). (A) Net and local drifts, (B) Age effect, (C) Period effect, (D) Cohort effect. RR: relative risk.

The net drift, all-age deviations, all-period deviations, all-period relative risk (RR), and all-cohort RR of the newly reported infections among older adults in Zhejiang Province from 2004 to 2021 were significant (all *P*<.001), indicating that changes in the newly reported infections were affected by age, period, and cohort factors (Table 3). As shown in Figure 3A, the net drift of the newly reported HIV infections was 31.501% (95% CI –25.510% to 37.780%). There was no significant change in local drift in the newly reported infections across age groups.

**Table 3:** Age-period-cohort analysis of the newly reported HIV infections among the older adults in Zhejiang Province.

	*χ*^2^ (*df*)	*P* value
Net drift=0	132.490 (1)	＜.001
All age deviations=0	26.946 (5)	＜.001
All period deviations=0	60.887 (2)	＜.001
All cohort deviations=0	3.537 (8)	.90
All period RR^a^=1	161.170 (3)	＜.001
All cohort RR=1	234.936 (9)	＜.001
All local drifts=net drift	3.485 (7)	.84

^a^RR: relative risk.

#### Age Effect

Figure 3B shows the newly reported HIV infections showed a monotonic increasing trend from 0.01 per 100,000 persons in the age group of 50-54 years to 16.71 per 100,000 persons in the age group of ≥80 years, and the rate of increase was significantly accelerated after the age of 70 years.

#### Period Effect

Using the 2007-2011 period group as a control group (RR=1), an overall upward trend was observed in the period effect of HIV/AIDS risk reported by older adults, with the RR increasing from 0.099 (95% CI –0.052 to 0.185) to 6.23 (95% CI –4.269 to 9.092; Figure 3C).

#### Cohort Effect

Using the 1940-1944 birth cohort as a control group (RR=1), the cohort effect on the risk of HIV/AIDS among older adults born before 1940 in Zhejiang Province did not change significantly. Since 1944, the birth cohort effect has increased monotonically, with the RR increasing from 4.134 (95% CI –2.999 to 5.700) in the 1945-1949 birth cohort to 1064.394 (95% CI –418.537 to 2706.892) in the 1965-1969 birth cohort (Figure 3D).

#### Spatial Analysis

#### Global Spatial Autocorrelation

Global spatial autocorrelation analysis of the newly reported infections for each year of the study period was performed and grouped according to different routes of infection (Multimedia Appendix 2). The results showed no significant differences in the global geographic autocorrelation of newly reported infections for the total population and the heterosexual transmission population. However, the global Moran’s *I* values for the newly reported rates of older MSM after 2012 were all greater than 0 (*P*<.05), indicating significant clustering of newly reported infections among older MSM in the Zhejiang Province. Therefore, further spatial clustering analyses are required in this population.

#### Local Spatial Autocorrelation

**Figure 4:**
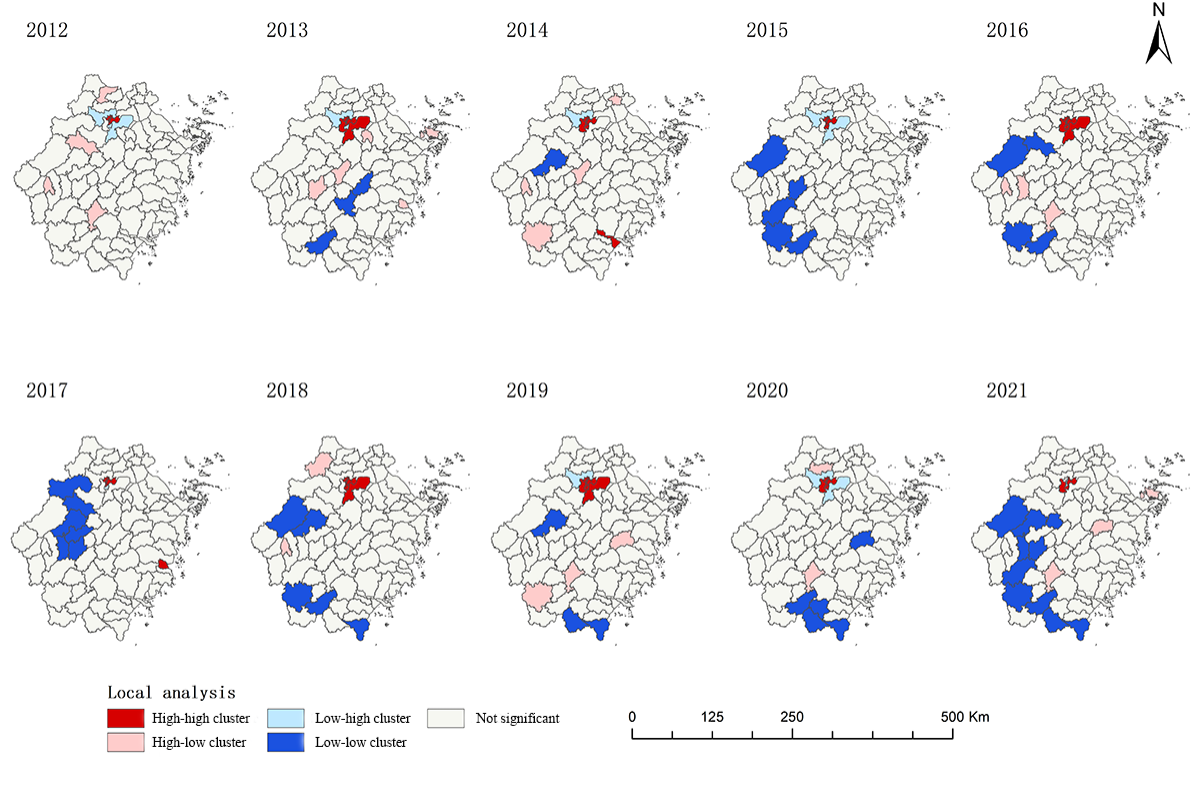
Local spatial autocorrelation analysis of HIV infection among older men who have sex with men in the Zhejiang Province.

LISA cluster distribution maps were used to identify HIV/AIDS hotspots and outlier sites among older MSM in the Zhejiang Province (Figure 4). In 2012, the high-high cluster areas of newly reported HIV infections among older MSM were concentrated in the urban areas of Hangzhou, including Shangcheng, Xiacheng, Gongshu, and Jianggan districts, and there were no low-low cluster areas. In the following years, the high-high cluster areas were also concentrated in the main urban area of Hangzhou, while the low-low cluster areas were small and unstable, scattered in Chun’an and Jiande counties as well as Cangnan and Taishun areas.

#### Spatial Regression Analysis

**Figure 5:**
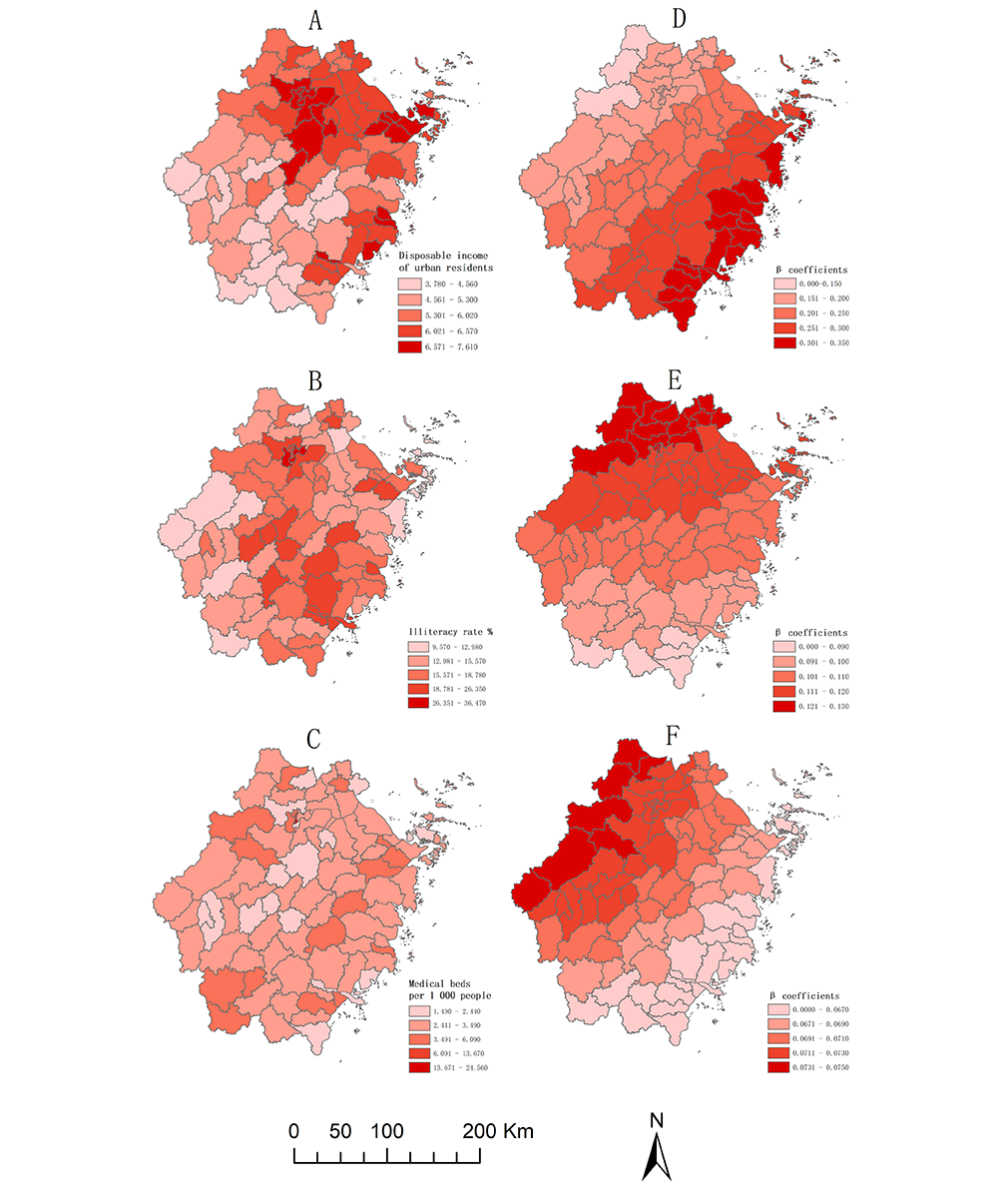
Spatial distribution of independent variables and regression coefficients.

In the GWR, the independent variables for the best model in 2020 include urban disposable income, the illiteracy rate of residents aged 15 years or older, and the number of hospital beds per 1000 people. Multimedia Appendix 3 shows that the GWR model is better than the OLS model and has no residual autocorrelation. Figure 5A-C shows the spatial distribution of the independent variables, including the disposable income of urban residents, the illiteracy rate of residents aged 15 years or older, and the number of hospital beds per 1000 people, respectively. Figure 5D-F shows the β coefficients determined using GWR regression, and Figure 5D indicates that the disposable income of urban residents in the Zhejiang Province is positively associated with the newly reported HIV infections among older MSM. The newly reported HIV infections were higher in cities with high urban disposable income in southeastern Zhejiang Province. In Zhejiang Province, the illiteracy rate of residents aged 15 years or older was positively associated with newly reported HIV infections in the population (Figure 5E). Figure 5F shows the newly reported HIV infections associated with the number of hospital beds per 1000 people; an increase in reported HIV infections in cities with a high number of hospital beds per 1000 people in northwestern Zhejiang Province was observed.

## Discussion

As HIV/AIDS is initially prevalent among individuals aged between 15 and 49 years, there are fewer reports of HIV infections in older adults [4]. The number and proportion of HIV/AIDS cases among older adults have increased significantly in recent years due to aging and the development of the HIV/AIDS epidemic. Yuan et al [8] found that the proportion of older adult cases of all reported HIV/AIDS cases in Sichuan Province, China, increased from 4.1% in 2008 to 59.2% in 2019. In the central and western regions of China, 98.2% of older adults were infected through heterosexual transmission [8], while in Zhejiang Province, 13.7% were infected through homosexual transmission. This suggests a slightly different pattern of HIV/AIDS prevalence in the eastern coastal region than in central and western China. However, few studies have used spatial and temporal distributions to analyze the prevalence of HIV/AIDS among older adults in the eastern, developed coastal areas of China. This study summarized the spatial and temporal distribution of the pandemic and its influencing factors, providing a rationale for designing prevention programs and allocating resources more efficiently.

Overall, the Joinpoint regression results showed that the proportion of newly reported HIV/AIDS cases increased significantly from 2004 to 2021. This phenomenon could be illustrated by several factors. First, the Chinese government adopted a policy of expanding testing in recent years, which has significantly improved the detection rate of the virus. This indicates that the expanded testing policy was widely implemented, leading to more HIV/AIDS being detected [19]. Second, at the beginning of the pandemic, HIV/AIDS mainly affected young individuals [20]; therefore, most education catered to that age group, while older individuals were overlooked, which increases their risk of infection in that age group. Third, the main route of HIV/AIDS transmission among older adults was heterosexual transmission (10,496/12,376, 84.8%). Low-cost commercial establishments are a major source of infection in older adults [21]. Simultaneously, older male individuals tend not to use condoms when engaging in heterosexual contact, which also increases their risk of HIV/AIDS transmission [4]. Finally, transportation, communication, and sexual activity have changed over the past decade with advances in technology and economic growth [8]. Access to transportation and communication has enhanced the sex work industry; with economic development, the older population has the financial means to pay for these services. This change also poses significant challenges to traditional HIV/AIDS prevention and control strategies [8]. Male individuals have significantly higher rates of HIV/AIDS than female individuals. Possible reasons for this are the lack of physiological decline in those older than 50 years of age and the high level of physical condition and sexual needs of older male individuals [22]. In summary, the current prevention services are inadequate for addressing the HIV/AIDS epidemic among older adults, and further research and public health interventions are required. Along with expanding testing, sex education for older adults should be strengthened to reduce the incidence of high-risk sexual behavior.

Age, period, and cohort had significant effects on newly reported HIV infections among older adults in Zhejiang Province. Age is an important factor influencing HIV/AIDS. Our findings suggest that, within the same birth cohort, older age correlates with a higher risk of reporting HIV/AIDS, which is consistent with previous results [23]. Lu et al [24] also found that HIV incidence among male individuals in Zhejiang Province peaked between the ages of 20 and 35 years, then declined and peaked again between the ages of 60 and 70 years. In this study, homosexual and heterosexual sexual contact were the main modes of HIV/AIDS transmission among older adults. Contrary to the common belief that sexual activity among older adults decreases with age, older adults still engage in sexual activity [25]. One study found that most older patients found sex with an HIV-positive partner to be acceptable [26]. However, unprotected sexual contact is common among older adults due to lower level of education, which increases their risk of HIV infection [4].

The period effect of newly reported HIV infections among older adults in the Zhejiang Province from 2004 to 2021 was consistent with the change in the Joinpoint regression. The monotonic upward trend in the period effect of newly reported infections was associated with factors such as policies to expand testing and unsafe sex among older adults [8,19,21]. Period effects are an important factor influencing HIV/AIDS transmission among older adults and is linked to medical advances and policy development. As economies and societies advance, multidisciplinary approaches to curb growing HIV/AIDS-related problems must be considered. For example, the Chinese CDC launched a web-based AIDS information system and integrated response information management system in 2006 [27]. This has implemented health promotion strategies within social networks and strengthened sexual health services and partner services.

Cohort effects included the risk of morbidity or mortality in individuals in the same birth cohort with the same exposure to a disease risk factor [24]. In this study, the birth cohort effect on newly reported HIV infections among older adults in Zhejiang Province increased from 2004 to 2021. Cohorts born before 1950 had fewer reported infections, whereas those born after 1950 showed a gradual increase in reported infections with increasing birth year. This may be attributed to the fact that the cohort born after 1950 is in the sexually active age group. A previous study reported higher rates of sexual transmission among people born later in life, which may indicate that they are more sexually active or risky [23].

Spatial analysis revealed a geographic expansion of the newly reported HIV infections among older adults in Zhejiang Province. HIV-positive MSM showed a spatial correlation during the study period. The spatial autocorrelation results showed high clustering of HIV-positive MSM in Hangzhou City. Factors influencing the aggregation of HIV infections include the local HIV burden [28]. The high number of chronically infected individuals in Hangzhou has hindered HIV/AIDS control. Sexual transmission has become a major mode of HIV transmission in Zhejiang Province; however, homosexuality remains highly stigmatized and discriminated against in China [29]. As a result, Chinese MSM tend to hide their identity and engage in sexual behavior in other areas. Most MSM are concentrated in metropolitan areas, where self-identification and sexual partners are available. Hangzhou is the capital city of the Zhejiang Province, with a rapidly growing economy that has attracted migrants, including MSM [30]. Meanwhile, the provincial transmission networks found that Hangzhou plays a central role in the cross-regional transmission of HIV/AIDS among MSM in Zhejiang Province [31]. Studies have found that stigma can also lead to risky behaviors, such as lack of testing and avoidance of treatment. Various forms of stigma, such as substance abuse, depression, and traumatic stress, interact to increase the vulnerability of MSM and increase the risk of HIV infection [32]. The stigma associated with MSM, coming out, accepting sexual partners, and being perceived as “older” in the gay community can lead to risky behaviors such as avoiding sex education [33]. This study provides clues to the epidemic clustering characteristics of older MSM in Zhejiang Province, which will facilitate the design and implementation of evidence-based interventions. Eliminating stigmatization and discrimination against MSM populations is critical to controlling epidemic aggregation and reducing HIV infection, as stigma and discrimination are barriers to MSM seeking HIV/AIDS services [32].

Overall, the GWR analysis showed that urban disposable income, the illiteracy rate of residents aged 15 years or older, and the number of hospital beds per 1000 people were associated with the reported infections of HIV-positive MSM among older adults in the Zhejiang Province. The economy is an important factor influencing the pattern of HIV-reported infections. People with a higher income and lower awareness of HIV/AIDS were more likely to be HIV-positive [18], and their high disposable income allowed them to have more sexual partnerships. Moreover, illiteracy was positively associated with the reported HIV infections among older MSM in Zhejiang Province. Higher educational attainment is associated with a lower risk of HIV infection; targeted educational programs and attitude changes among people living with HIV/AIDS can help advance voluntary counseling and testing [34]. This study demonstrates the need to provide health education for older adults to further promote sexual health in older adults. Finally, the number of hospital beds per 1000 people was strongly associated with the reported infections among older MSM in Zhejiang Province. Health care is an important determinant of HIV/AIDS prevention. Qin et al [35] found a significant positive association between the number of health care facilities and the number of reported cases of HIV/AIDS. Detection bias is a possible cause, and better health care resources are associated with higher detection rates; therefore, areas with abundant health care resources generally have more diagnosed cases. The provision of good health care is strongly associated with early diagnosis of HIV/AIDS, which can help control the spread of the epidemic.

This study has some limitations. First, since this was an ecological study, it was difficult to determine the causal relationships between the outcomes and variables. Second, the number of newly reported cases differs from the incidence, and the number of reported HIV/AIDS cases among older adults is influenced by the testing intensity, coverage, and efficiency. The number of HIV/AIDS tests decreased by 41% worldwide due to inadequate HIV/AIDS testing in health services as a result of the COVID-19 pandemic [36]. In Zhejiang Province, where the number of reported cases among older adults began to decline for the first time in 2019, future research must assess the impact of the COVID-19 pandemic on HIV/AIDS testing. Third, spatial autocorrelation is influenced by partition effects. In this study, we used county districts as the spatial units of analysis. Different spatial statistical values would have been obtained had the analysis been conducted at the municipal or street levels. Further studies should consider using smaller spatial units for analysis, which may provide more locational information and make the results more comprehensive. Fourth, the GWR model used indicators obtained from the local bureau of statistics, which did not have specific indicators for older people. However, we have attempted to use these indicators to reflect the overall level of development in the area. Despite these limitations, we identified an increasing trend of HIV/AIDS among older adults in the Zhejiang Province, along with spatial clustering in the epidemic distribution.

In summary, the overall number of newly reported HIV infections among older adults in Zhejiang Province has increased in recent years, and older adults have become a key population in the HIV/AIDS epidemic. The majority of HIV/AIDS among older adults is transmitted through heterosexual transmission, but cases among MSM are showing aggregation in some counties. The combination of economic growth and low cognition among older adults are positively correlated with reported HIV infections, so prevention and control strategies should be more inclined to focus on the lower literacy level of older populations in better-off areas. Finally, areas with better health care resources can increase detection; therefore, it is important to comprehensively evaluate testing systems in different regions and improve access to testing services.

## Appendix

Multimedia Appendix 1 Location of the study area, Zhejiang Province, China.

Multimedia Appendix 2 Spatial autocorrelation of reported HIV infections among older adults in Zhejiang Province, 2004-2021.

Multimedia Appendix 3 Results of the selected explanatory model in ordinary least squares and geographically weighted regression.

## Abbreviations

AAPC: average annual percent change
APC: annual percent change
CDC: Center for Disease Control and Prevention
GDP: gross domestic product
GWR: geographically weighted regression
LISA: Local Indicators of Spatial Autocorrelation
MSM: men who have sex with men
OLS: ordinary least squares
RR: relative risk

## Data Availability

The data sets generated and analyzed during this study are available from the corresponding author on reasonable request.
