# Supplementary figures and images for: Spatiotemporal Pattern and Its Determinants for Newly Reported HIV/AIDS Among Older Adults in Eastern China From 2004 to 2021: Retrospective Analysis Study
Source: JMIR Public Health Surveill. 2024 Feb 13;10:e51172. doi: 10.2196/51172 (PMC10900086; doi:10.2196/51172)

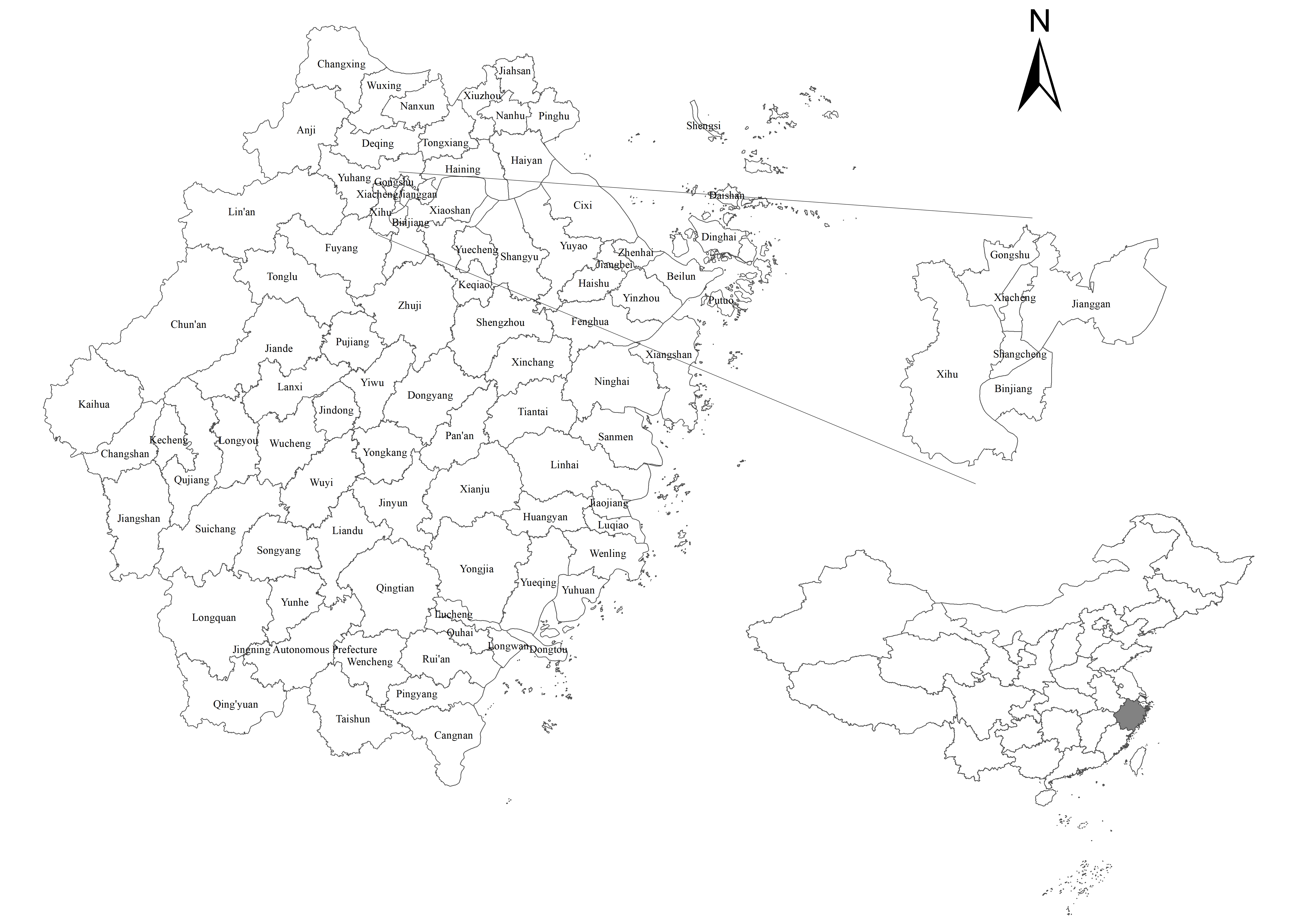

Supplement: Multimedia Appendix 1 [file publichealth_v10i1e51172_app1.png]
