# Supplementary material for: Spatiotemporal Pattern and Its Determinants for Newly Reported HIV/AIDS Among Older Adults in Eastern China From 2004 to 2021: Retrospective Analysis Study
Source: JMIR Public Health Surveill. 2024 Feb 13;10:e51172. doi: 10.2196/51172 (PMC10900086; doi:10.2196/51172)
Supplement: Multimedia Appendix 2 [file publichealth_v10i1e51172_app2.docx]

Multimedia Appendix 2. Spatial autocorrelation of reported HIV/AIDS incidence among older adults in Zhejiang Province, 2004-2021.

| Route of transmission | Year | ***Moran’ I*** | ***Z score*** | ***P Value*** |
| --- | --- | --- | --- | --- |
| Overall | 2004 | -0.0535 | -0.7028 | 0.4822 |
|  | 2005 | 0.2400 | 3.9316 | 0.0001 |
|  | 2006 | 0.1017 | 1.6658 | 0.0958 |
|  | 2007 | -0.0333 | -0.3164 | 0.7517 |
|  | 2008 | 0.0281 | 0.6256 | 0.5316 |
|  | 2009 | 0.0945 | 1.8663 | 0.0620 |
|  | 2010 | 0.0614 | 1.1375 | 0.2553 |
|  | 2011 | 0.1113 | 2.0256 | 0.0428 |
|  | 2012 | 0.0536 | 1.0584 | 0.2899 |
|  | 2013 | 0.0893 | 1.5522 | 0.1206 |
|  | 2014 | 0.0615 | 1.1022 | 0.2704 |
|  | 2015 | 0.0442 | 0.9096 | 0.3630 |
|  | 2016 | 0.1112 | 1.8637 | 0.0624 |
|  | 2017 | 0.0376 | 0.7929 | 0.4278 |
|  | 2018 | 0.0277 | 0.6204 | 0.5350 |
|  | 2019 | 0.1079 | 1.7820 | 0.0747 |
|  | 2020 | 0.0607 | 1.0955 | 0.2733 |
|  | 2021 | 0.0362 | 0.6856 | 0.4930 |
| Heterosexual | 2004 | -0.0500 | -0.6779 | 0.4979 |
|  | 2005 | 0.1568 | 3.1751 | 0.0015 |
|  | 2006 | 0.1118 | 1.8108 | 0.0702 |
|  | 2007 | -0.0555 | -0.6448 | 0.5191 |
|  | 2008 | 0.0397 | 0.7780 | 0.4365 |
|  | 2009 | 0.0916 | 1.6092 | 0.1076 |
|  | 2010 | 0.0792 | 1.3149 | 0.1885 |
|  | 2011 | 0.1261 | 1.9899 | 0.0466 |
|  | 2012 | 0.0512 | 0.9945 | 0.3200 |
|  | 2013 | 0.0276 | 0.5551 | 0.5788 |
|  | 2014 | -0.0290 | -0.2704 | 0.7869 |
|  | 2015 | -0.0011 | 0.1476 | 0.8827 |
|  | 2016 | 0.0286 | 0.5698 | 0.5688 |
|  | 2017 | -0.0036 | 0.1375 | 0.8906 |
|  | 2018 | 0.0082 | 0.2842 | 0.7763 |
|  | 2019 | 0.0783 | 1.3140 | 0.1888 |
|  | 2020 | 0.0581 | 1.0414 | 0.2977 |
|  | 2021 | -0.0186 | -0.1056 | 0.9159 |
| Homosexual | 2006 | -0.0194 | -0.2103 | 0.8334 |
|  | 2007 | -0.0133 | -0.3623 | 0.7171 |
|  | 2008 | 0.0386 | 0.7951 | 0.4266 |
|  | 2009 | -0.0250 | -0.2001 | 0.8414 |
|  | 2010 | 0.1435 | 4.0925 | ＜0.0001 |
|  | 2011 | 0.0750 | 1.4340 | 0.1516 |
|  | 2012 | 0.2390 | 4.1379 | ＜0.0001 |
|  | 2013 | 0.2727 | 4.1891 | ＜0.0001 |
|  | 2014 | 0.3766 | 5.6292 | ＜0.0001 |
|  | 2015 | 0.2700 | 4.6508 | ＜0.0001 |
|  | 2016 | 0.2776 | 4.2802 | ＜0.0001 |
|  | 2017 | 0.2083 | 3.2243 | 0.0013 |
|  | 2018 | 0.2453 | 4.3213 | ＜0.0001 |
|  | 2019 | 0.2459 | 3.8607 | 0.0001 |
|  | 2020 | 0.3162 | 4.8870 | ＜0.0001 |
|  | 2021 | 0.1158 | 2.1722 | 0.0298 |
