# Supplementary material for: Spatiotemporal Pattern and Its Determinants for Newly Reported HIV/AIDS Among Older Adults in Eastern China From 2004 to 2021: Retrospective Analysis Study
Source: JMIR Public Health Surveill. 2024 Feb 13;10:e51172. doi: 10.2196/51172 (PMC10900086; doi:10.2196/51172)
Supplement: Multimedia Appendix 3 [file publichealth_v10i1e51172_app3.docx]

Multimedia Appendix 3. Results of the selected explanatory model in OLS and GWR.

| Analysis | Model | |
| --- | --- | --- |
|  | OLS | GWR |
| AICc | 209.5698 | 208.3053 |
| R2 | 0.4660 | 0.4936 |
| Adjusted R2 | 0.4471 | 0.4589 |
| Residues Moran’s Index | 0.0066 | -0.0267 |
| z-score | 0.2613 | -0.2239 |
| p-value | 0.7938 | 0.8228 |

Legend: AICc, corrected Akaike information criterion; OLS, ordinary least squares; GWR, geographically weighted regression.
